# Exploratory study on classification of lung cancer subtypes through a combined K-nearest neighbor classifier in breathomics

**DOI:** 10.1038/s41598-020-62803-4

**Published:** 2020-04-03

**Authors:** Chunyan Wang, Yijing Long, Wenwen Li, Wei Dai, Shaohua Xie, Yuanling Liu, Yinchenxi Zhang, Mingxin Liu, Yonghui Tian, Qiang Li, Yixiang Duan

**Affiliations:** 1 Research Center of Analytical Instrumentation, Key Laboratory of Bio-source and Eco-environment, Ministry of Education, College of Life Sciences, Sichuan University, Chengdu, 610064 P.R. China; 2 West China School of Public Health and West China Fourth Hospital, Sichuan University, Chengdu, 610041 P.R. China; 3 Department of Thoracic Surgery, Sichuan Cancer Hospital & Institute, Sichuan Cancer Center, School of Medicine, University of Electronic Science and Technology of China, Chengdu, Sichuan China; 4 Graduate School, Chengdu Medical College, Chengdu, Sichuan China; 5 College of Chemistry and Material Science, Northwest University Department of Chemistry and Material Science, Xi’an, 710127 Shanxi Province P.R. China

**Keywords:** Cancer, Prognosis

## Abstract

Accurate classification of adenocarcinoma (AC) and squamous cell carcinoma (SCC) in lung cancer is critical to physicians’ clinical decision-making. Exhaled breath analysis provides a tremendous potential approach in non-invasive diagnosis of lung cancer but was rarely reported for lung cancer subtypes classification. In this paper, we firstly proposed a combined method, integrating K-nearest neighbor classifier (KNN), borderline2-synthetic minority over-sampling technique (borderlin2-SMOTE), and feature reduction methods, to investigate the ability of exhaled breath to distinguish AC from SCC patients. The classification performance of the proposed method was compared with the results of four classification algorithms under different combinations of borderline2-SMOTE and feature reduction methods. The result indicated that the KNN classifier combining borderline2-SMOTE and feature reduction methods was the most promising method to discriminate AC from SCC patients and obtained the highest mean area under the receiver operating characteristic curve (0.63) and mean geometric mean (58.50) when compared to others classifiers. The result revealed that the combined algorithm could improve the classification performance of lung cancer subtypes in breathomics and suggested that combining non-invasive exhaled breath analysis with multivariate analysis is a promising screening method for informing treatment options and facilitating individualized treatment of lung cancer subtypes patients.

## Introduction

Lung cancer is one of the most malignant tumors threatening people’s health and life, which is divided into small cell lung cancer (SCLC, ~15%) and non-small cell lung cancer (NSCLC, ~85%), NSCLC mainly includes adenocarcinoma (AC, ~38%) and squamous cell carcinoma (SCC, ~20%)^1,2^. Numerous clinical trials have proved that the more exact the type of tumor histology we know, the more effective treatment would be^1,3^. Therefore, how to quickly distinguish the exact subtypes of lung cancer, especially AC and SCC, has become a mandatory diagnostic requirement in the past decades years^4–6^. In clinical practice, the histopathological analysis is a gold standard for diagnosing the lung cancer subtypes, but it can cause invasive injury and is complicated in operation. Another common method is imaging diagnosis (e. g., low-dose computed tomography (LD-CT)), which has certain limitations of radiation exposure and high false-positive rate. Thus, the non-invasive and safe alternative diagnosis methods based on omics analysis (e. g., proteomics and radiomics) are pursued by experts to distinguish lung cancer subtypes^6–12^.

Similar to other omics, breathomics as a non-invasive diagnostic method had shown its potential for early diagnosis of diseases, prognosis evaluation, and classification of disease subtypes^13,14^. Relative studies also revealed the possibility of machine learning algorithms coupled with high-throughput platforms to the classification problem of lung cancer subtypes *in vitro*^15^. In 2012, Barash *et al*. proved that combining support vector machines with gold nanoparticle sensors and gas chromatography coupled with mass spectrometry (GC-MS) can significantly discrimination between AC and SCC in the headspace of lung cancer subtypes cells^1^. Santonico *et al*. and his group explored the effect of two breath sampling method on classification accuracy of AC and SCC after combining GC-MS and gas sensor array for exhaled breath analysis. Their results claimed that the classification performance of endoscopic breath sampling (EBS) outperformed than bag breath sampling (BBS) and could achieve 75% accuracy. Unfortunately, given the complexity of the sampling, they only performed the gas analysis in small size sample^16^. In the same year, Mazzone *et al*. demonstrated the feasibility of classifying the exhaled breath of lung cancer patients with different histological subtypes through a colorimetric sensor array. They obtained excellent classification results using a backward step-down feature selection method before performing a logistic regression analysis. Nevertheless, it was regrettable that they were unable to qualitatively analyze the features due to the dye-analyte interaction principle of colorimetric sensors^17^. Recently, Handa *et al*. found the feasibility of decision trees algorithm for the identification of AC and SCC using ion mobility spectrometry. However, their classification results did not present details results about diagnostic ability, specificity, and sensitivity^18^. Most of the studies in breathomics analysis used algorithms alone to achieve the goal of distinguishing tumor histological subtypes, and they pay less attention to data pre-processing of the breathomics data to obtain exact information before conducting the regression or classification model. Studies have proven that data pre-processing is an indispensable part of multivariate analysis^19,20^.

Data pre-processing is a series of operations before classification tasks, including outlier exclusion, missing data imputation, feature project, class balance. In this study, we will detail discuss the feature engineering and class balance, which are the most critical factors affecting the performance of the classification model. The breathomics data generated by the high-throughput analytical platforms contain redundant information such as mixture interference in the exhaled breath. Hence, how to select the data features and build an effective and simple model should be considered. Feature extraction or selection methods can not only reduce the risk of over-fitting by removing irrelevant features but also improve the accuracy of the classification model^21,22^. Besides, the classification task of lung cancer subtypes is always faced with the problem of imbalanced data distribution. The data imbalance has a negative impact on the performance of the classification model in the training phase and can lead to a high false-positive rate^23^. Nowadays, resampling techniques have gradually been adopted in unbalanced data processing because of its simplicity and ease of implementation^24–26^.

In this study, we collected 325 exhaled breath samples (including 234 AC and 91 SCC patients), and the volatile organic compounds (VOCs) were analyzed by GC-MS analysis. In consideration of the barriers of unbalanced proportion and information redundancy in data, a combined method based on the K-nearest neighbor (KNN) algorithm was proposed for better classification performance. With the help of resampling technology and feature dimensionality reduction methods, we can exactly extract important breathomics features, to improve classification model performance of lung cancer histological subtypes. Additionally, the widely used analysis algorithm, such as partial least squares-discriminant analysis (PLS-DA)^27^, the random forest (RF)^28^, support vector machine (SVM)^1^, and multilayer perceptron network (MLP)^12^, were performed for comparison in this study. The results demonstrated the potential of a combined KNN classifier for the prediction of lung cancer subtypes. The proposed method would be a powerful tool to assist physicians in drawing a better decision during the diagnostic procedure. In the future, we would pay more attention to data mining and design the algorithm structure for an efficient diagnosis in this field.

## Materials and methods

### Subjects and exhaled breath sampling

All individuals are newly admitted patients and have a pathological diagnosis of primary lung cancer, according to the 2015 World Health Organization Classification of lung tumors. Moreover, the subtypes (AC or SCC) of the patients are confirmed based on a postoperative pathological examination. All volunteers signed informed consent after a detailed introduction of this study. This study was approved by the Ethics Committee of Sichuan Cancer Hospital. All methods were carried out in accordance with the guidelines and regulations of the Good Clinical Practice Guidelines and the Declaration of Helsinki. Our exhaled breath collection method was guided by referring to the previous studies^15,29^. Participants underwent overnight fasting for at least 8 hours and rest in a well-ventilated, separate room for at least 10 minutes before breath sampling in the morning. Subjects were required to have normal breathing before collection. Deep inhalation and nasal ventilation were not allowed during sampling in case of ambient air dilution. Breath samples were collected by Bio-VOC breath sampler (Markes Int. the U.K) to exclude the dead space air from the oral and upper respiratory tract. The end-tidal breath gases were retained in the Bio-VOC syringe, and then be transferred to the Tedlar bag (500 mL) through a three-way valve. The exhaled breath samples were stored at −40 °C until analysis (within one week).

### Exhaled breath analysis

The samples were analyzed using gas chromatography-mass spectrometry (GC-MS) (GC 1300; MS TSQ8000; ThermoScientific; America) in split mode (5:1) with a DB-624UI capillary column (60 m × 0.25 mm × 0.25 μm). Helium (99.99%) was used as the carrier gas at a constant flow rate of 1.0 mL·min^−1^. The compounds in exhaled breath samples were pre-concentrated by solid-phase micro-extraction (SPME) fibre of divinylbenzene/carboxy/polydimethylsiloxane-coated (DVB/CAR/PDMS) for 30 minutes at 37 °C. Then the SPME fibre was desorbed for 5 minutes in the GC front inlet. The temperatures of the GC front inlet, transfer line, and MS ion source were 270 °C, 250 °C, and 250 °C, respectively. Electron ionization (EI) source (70 eV) was used in MS. The MS detector was set in full scan mode with a mass range of 35–250* m/z* and a scan rate of 0.5 scan·s^−1^. The programed-temperature was set as follows: initially at 40 °C for 5 min, then increased to 160 °C at 10 °C·min^−1^, then to 200 °C at 5 °C·min^−1^, and kept for 15 min.

### Data processing methods

The raw data of GC-MS were transformed into the mzXML format files by ProteoWizard 3.0. The eRah package based on R language used a moving-minimum filter and a Savitzky-Golay filter to correct baseline drift and remove noise in chromatograms; then a two-step compound deconvolution was performed (minimum compound peak width is set at 5); finally, alignment (minimum spectral similarity and maximum retention time drift is set at 0.4 and 3 seconds respectively) and missing compounds recovery (minimum samples = 130) were carried out to correct retention time variation and kept the most of information in chromatograms^30^. The missing compounds recovery means the numbers of a feature appears in all samples. Here, we kept the features in 40% samples to get more breathomics features. The compounds, including the column bleeding and air pollutants, were excluded. Thus, 109 breathomics features were identified in each sample as our research objects, resulting in a 109 × 325 data matrix.

#### Outliers detection in multivariate analysis

The data points (AC and SCC samples) whose values do not accord with the vast majority samples are considered as outliers. The peaks of these data points need to be re-examined in the original chromatogram and perhaps require discarding the data points^31^. In general. Outliers can be divided into two types: biological or analytical outliers. The biological outliers are difficult to identify, and it occurs due to random or induced biological variation among samples^32^. However, the analytical outliers generated in the process of sampling, storage, pretreatment, sample analysis are easy to be detected and need to be excluded owing to their serious distortion of biological information. In univariate analysis, box-plot analysis is often used to detect outliers, but not applicable to outliers of more than two variables (features). Principal component analysis (PCA) is an effective method for recognizing outliers in multivariate analysis and can get rid of the prior knowledge for the original data. Outliers can be detected by using the Hotelling’s T2 range in PCA, which is measured by calculating the distance between each sample and the center of the samples^33^. The Hotelling’s T2 range of a sample exceeding the critical threshold (95% or 99% T2Crit) means that the sample is far from the other samples in the PCA score-space. Thus it should be excluded since the probability of it belongs to the same class as other samples are lower than 5% or 1%. After the analytical outliers were eliminated, the range scaling method was used to normalize each breathomics feature to a range of 0 to 1 before further analysis^34^.

#### Resampling techniques

Over-sampling and under-sampling methods may lead to unsatisfactory classification performance since these methods replicate existing samples in the minority class or reduce samples in the majority class to balance the proportion of classes^26,35^. Thus, the variant of synthetic minority over-sampling technique (SMOTE) like borderline-SMOTE is proposed to achieve more realistic classification prediction by resampling the borderline data of minority class as exactly as possible in the training phase^23,36,37^. In the process of the borderline-SMOTE, we first calculate the nearest neighbors (m) of each SCC sample p_i_ (i = 1, 2, …, n) from the whole training set (minority class is SCC patients, and majority class is AC patients). m′ represents the number of neighbors belonging to the AC class among the m nearest neighbors. If m′ = m, p_i_ is considered to be noise, and if 0 ≤ m′ < m/2, p_i_ is a safe sample, under both situations, the p_i_ does not need to participate in the resampling process. If m/2 ≤ m′ < m, p_i_ is easily misclassified and is deemed a danger sample. Then, Borderline-SMOTE only resamples danger samples, which are the borderline data in the minority class. S_j_ is the new synthetic minority sample and is generated by the function S_j_ = p_i_′ + r_j_ × dif_j_ (j = 1, 2, …, s). Where p_i_′ (i = 1, 2, 3, …, n) is the danger sample set, r_j_ is a random number between 0 and 1, dif_j_ represent the differences between p_i_′ and its nearest neighbors (randomly select s nearest neighbors) from SCC samples. Final, the minority class samples is P′ = P + S_j_. We can know that the new sample set is generated along the line between the minority borderline samples and their nearest neighbors of the same class. In this work, borderline2-SMOTE is considered as our class balance method, and the random number r_j_ is set as between 0 and 0.5 by referring to the literature; thus the newly generated samples are closer to the minority class^37^.

#### Feature dimensionality reduction methods

The number of breathomics features may increase the complexity of the calculation in classification algorithms, especially in RF, and KNN classifiers^38^. Therefore, feature extraction and feature selection methods are applied to reduce irrelevant features. In general, Feature extraction method is widely used in omics analysis to obtain the linear combination of original features by changing feature space, like the linear feature extraction methods (e.g., PCA, LDA) and non-linear feature extraction methods (e.g., manifold learning and kernel PCA)^39^. PCA is popular for exploring and reducing multidimensional data in omics analysis^19^. The theory of the PCA algorithm is: the eigenvalues of the covariance matrix of the original dataset were arrayed from large to small as: λ_1_, λ_2_, …, λ_n_. By selecting different eigenvalues n, the transformed features under different principal components can be obtained. Besides, PCA can also be used for data visualization exploration in a two-dimensional or three-dimensional manner. The main shortcoming of PCA is that the principal component extracted is the combination of original features and the important features separating samples of different categories cannot be found indirectly.

Compared with the feature extraction method based on projection or compression, the feature selection method reduces the feature dimensions by selecting feature subsets instead of transforming them. Feature selection methods primarily include the filter, wrapper, embedded method based on how they interact with classifiers^22^. The wrapper method was adopted in our work because of its popularity and high computational efficiency in the reported literature^21^. In a wrapper method, the feature reduction process is related to the base classifier, and its performance is used as an evaluation criterion for feature selection. The method of combining linear support vector machines (SVM) with feature selection backward elimination is called SVM-recursive feature elimination (SVM-RFE)^40^. Linear SVM assigns weights (w) to each feature, and the goal of RFE is to select feature subsets by recursively considering smaller and smaller subsets of features. Firstly, the SVM is trained on the initial set of features and weights are assigned to each one of them; then features whose absolute weights are the smallest are pruned from the current features subset; final, the above procedure is recursively repeated on the pruned subsets until the desired number of features is reached. Compared with other feature selection methods, SVM-RFE is a scalable and efficient wrapper method^41,42^.

#### Supervised classification algorithms

All of the supervised classifiers are based on accepted advantages, but each approach has specific limitations. The frequently-used supervised classification algorithms are considered to perform the classification of lung cancer subtypes in our work. PLS-DA is a PLS regression where Y is a set of binary variables describing the categories of a categorical variable on data X. It establishes a regression model between the independent variables X and the classification variables Y of the training sample to effectively extract the feature variables related to classification. Although PLS-DA can reduce the dimension and noise in the matrix of independent variables, it can only fit the linear classification problems and is easily caught in the over-fitting problem^43^. The following classifiers can be used for nonlinear classification tasks. KNN uses distance measure to compare each new sample with the existing sample, and predicts the unlabeled sample category by the voting mechanism^44^. The advantage of KNN is easy to understand, implement, and can produce accurate results when the appropriate neighbors are selected. RF is an ensemble algorithm that integrates bagging technology and decision tree algorithm^45^. Its core idea is to use random return sampling rules to perform n sampling from the original data of N samples to form a training set containing n samples. It needs repeat sampling and establishes the decision tree model and determine the new sample category through multiple models. RF classifier is resistance to the different types of outliers, mislabeled samples and shows high performance for classification^19^. Non-linear SVM is widely used to classify the biological datasets. Typically, data point coordinates are transformed into a higher dimensional coordinate space through a kernel function where the SVM can draw a flat boundary between the transformed datasets^46^. SVM has been proved to be very insensitive to perturbations and outliers, especially for large sample size datasets since only a few support vector points affect the boundary^47^. MLP is a relatively simple feed-forward network. Its connectivity is similar to biological neural circuits, which include the input layer, hidden layer, and output layer. Each neuron in an MLP performs a weighted sum of its input and transforms it through a nonlinear activation function in the hidden layer, typically a squashing sigmoidal, and final export to the output layer. The complexity of MLP can be controlled by limiting the network size and constraining neuron weight values to avoid over-fitting^48,49^. These supervised classifiers have been applied to the classification of lung cancer subtypes in breath analysis, for instance, for analyzing sensor array data^17^, and mass spectrometry data due to their ease of implementation and high efficiency^18^.

### Implementation and evaluation of classification algorithms

**Figure 1:**
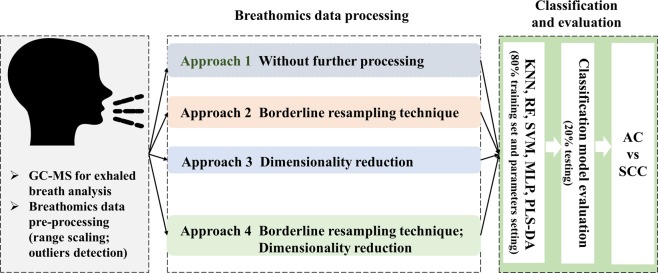
Study design flow. The input data first is processed using approach 1: without any processing; approach 2: borderline resampling technique only; approach 3: dimensionality reduction only; approach 4: dimensionality reduction and borderline resampling technique. And then, five classifiers are applied to establish a classification model in the training phase; final, the classification performance is evaluated in the testing set.

The workflow of supervised classification analysis is illustrated in Fig. 1. Only outlier detection was completed in SIMCA-P (version 13.0), and the implementation of multivariate analysis depended on the Python (version 3.6.2). The original data after pre-processing (raw spectrometry information extractions, outlier detection, and scaling range) was split into The Ethics Committee approved all procedures performed in this work involving human participants80% training set and 20% testing set. The 80% training set was used to establish the classification model and perform borderline2-SMOTE. in approaches 2 and 4, the nearest neighbor K in the borderline2-SMOTE is set as 5, and each classification model was repeated three times to decrease the randomness in the process of borderline2-SMOTE. For each classification model in approaches 1 and 2, the neighbors (K = 1, 2, …, 100) for KNN, the numbers of estimator (N = 5, 15, …, 300) for RF, and the number of neural M (M = 3, 4, …, 20) for MLP in hidden layer were selected using inner 5-fold cross-validation in the training test. The penalty parameter C (C = 1, 2, …, 30) and kernel function parameter gamma (gamma = 1/100, 2/100, …, 31/100) were optimized by using a grid search algorithm. Especially, the 80% training set and 20% testing set was used to find the optimal number of components n (n = 2, 3, …, 10) for PLS-DA model. PCA was used to visualize the class distribution of breathomics data before and after the execution of borderline2-SMOTE in the training set. In approaches 3 and approach 4 (with resampling technique), we incrementally selected 5–50 features or components in SVM-RFE or PCA with an increment of 5 features (5, 10, …, 50) to establish classification models in the training set and then the same features selected on the training set were chosen on the testing set. Final, the selected features were used for subtype classification with five classifiers using default parameters (K = 5 for KNN; N = 10 for RF; C = 1, and gamma = 0.1 for SVM; M = 100 for MLP; n = 2 for PLS-DA).

The evaluation of classification models was completed in the 20% testing set. In this work, AC is considered as the positive class. Sensitivity, specificity, the area under the receiver operating characteristic curve (AUC), and geometric mean (G-mean) are used to evaluate the predictive performance of classification models comprehensively. Sensitivity and specificity represent true positive rate and true negative rate, respectively as written in Eqs. (1) and (2). Where FN is defined as a false negative and FP as a false positive, TP and TN are defined as a true positive and a true negative, respectively. AUC is the curve area of true positives as a function of false positives^50,51^. G-mean measures the ability of the model to correctly classify negative and positive classes, which evaluates the overall performance of the classification model. The mathematical formula of G-mean is written as the following Eq. (3). In general, the sensitivity, specificity, AUC, G-mean value correspond to better predictive performance.

1 $${\rm{Sensitivity}}=\frac{{\rm{TP}}}{{\rm{TP}}+{\rm{FN}}}$$

2 $${\rm{Sensitivity}}=\frac{{\rm{TN}}}{{\rm{FP}}+{\rm{TN}}}$$

3 $${\rm{G}}-{\rm{mean}}=\sqrt{{\rm{Sensitivity}}}\times \sqrt{{\rm{Specificity}}}$$

### Ethical declaration

The Ethics Committee of Sichuan Cancer Hospital on April 6, 2017 (No.SCCHEC-02-2017-011) approved all procedures performed in this work involving human participants.

## Results

### Principal components analysis for outlier detection in lung cancer subtypes dataset

**Figure 2:**
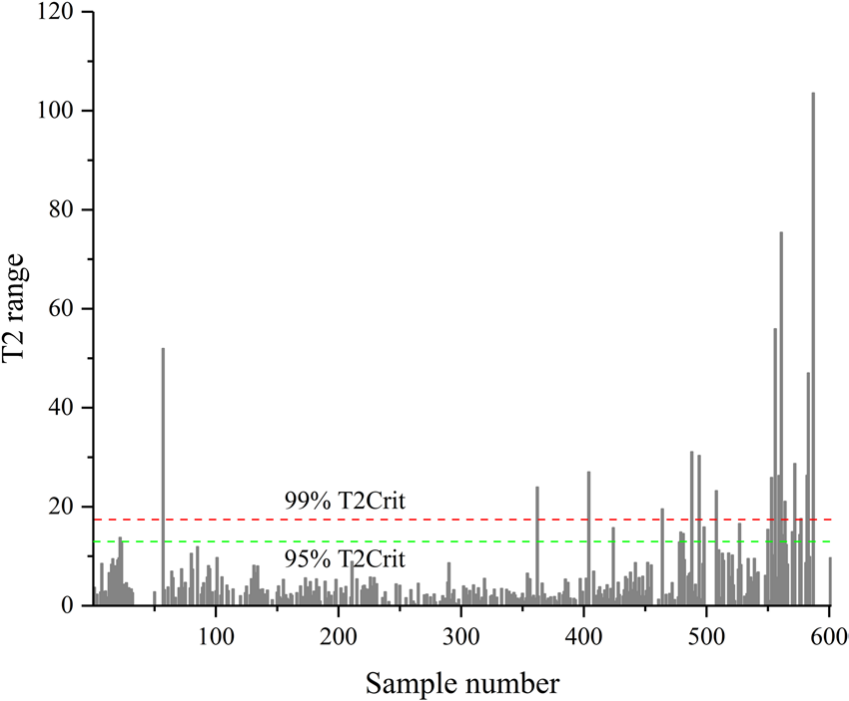
The Hotellingss T2 range is plotted for outlier detection by the sample number on the horizontal axis and T2 range on the vertical. The green and red dotted lines represent the 95% and 99% confidence intervals, respectively.

Table 1 summarized the demographic information of the AC and SCC subjects enrolled in this study. Exhaled breath samples were collected from AC (n = 234) and SCC (n = 91) patients. We have known that the incidence of AC was similar in men and women, while the incidence of SCC was higher in men in our work. Moreover, the proportion of lung cancer subtypes in the early and advanced stage were close to the same. One hundred and nine breathomics features were detected after excluding the pollutants. The principal component analysis (PCA) was first used to identify outliers without making any assumptions about the distribution of data. In the PCA score scatter plot (available in SIMCA-P 13.0), the components are set as 6 by using ‘Autofit’. In order to eliminate extremely outliers, we set the point far away from the elliptic border representing the 99% confidence intervals as a strong outlier. Furthermore, in our result, the outliers were the points whose score of T2 range was above the 17.422 (T2 range with 99% confidence intervals) by observing the ‘Hotelling’s T2 in Fig. 2. Therefore, thirteen AC and 3 SC samples were excluded and finally formed a 109 × 309 data matrix.

**Table 1:** Demographics of adenocarcinoma patients and squamous cell carcinoma patients. Data are expressed as mean ± standard deviation for age and BMI.

Clinical parameters	Adenocarcinoma	Squamous cell carcinoma
Sex		
Male	125 (53.42%)	88 (96.70%)
Female	109 (46.58%)	3 (3.30%)
Age		
Mean±SD	61 ± 7.09	62 ± 6.72
BMI		
Mean±SD	23.67 ± 3.03	22.48 ± 3.05
Smoking status		
Smoker	67	51
Non-smoker	136	13
Stopping smoker	31	27
Drinking status		
Drinker	48	24
Non-drinker	167	24
Stopping drinker	19	43
Education status		
Primary school	92	39
High school above	116	45
None	26	7
TNM stage		
I	110 (47.01%)	16 (17.58%)
II	24 (10.26%)	25 (27.47%)
III	45 (19.23%)	35 (38.46%)
IV	55 (23.50%)	15 (16.48%)

### The classification performance of lung cancer subtypes with resampling only

**Figure 3:**
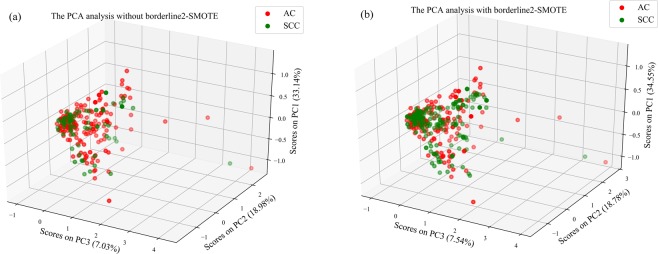
The visualization of the 3D scatters plot (**a**) before and (**b**) after the process of borderline2-SMOTE in PCA. These three axes represent the first three principal components. Abbreviations: PC: principal component. AC (red circle) and SCC (green circle) represent adenocarcinoma and squamous cell carcinoma, respectively.

The minority class (SCC) samples account for about 28% in approach 1. We resampled the SCC samples and made the class ratio of AC (n = 175) to SCC (n = 172) to be almost equal in the training set by using borderlin2-SMOTE in approach 2. The class distribution of the training data, before and after resampling, was shown in Fig. 3. As can be seen in the PCA scatter plot (Fig. 3), the data in the SCC class was noticeably increased after resampling, but not yet for classification trends between SCC and AC patients. Thus supervised classifiers were utilized to get better classification performance.

**Figure 4:**
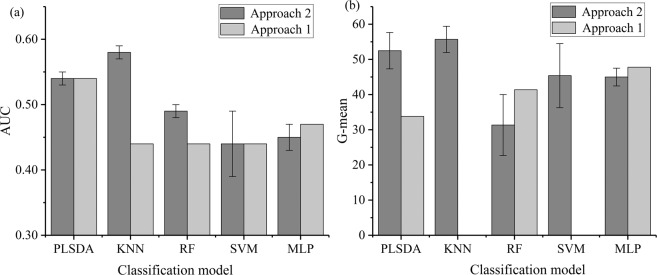
The classification result of G-mean and AUC value in five classifiers. (**a**,**b**) Represent the results of AUC and G-mean, respectively. Error bars are added to approach 2 for considering the average result after borderline2-SMOTE.

The performance of five different supervised classifiers in approach 1 and the average±standard deviation (SD) in approach 2 were presented after parameter optimization (Table 2). The results in Table 2 indicated that the sensitivity and specificity of models were relatively balanced after resampling, and not like that in approach 1 with very high sensitivity but poor specificity. High sensitivity meant the more AC samples were correctly classified, while poor specificity (Specificity = 0) represented the SCC samples were completely misclassified. The AUC and G-mean values of models were shown in Fig. 4, what can be seen is that the overall performance of classifiers has been significantly improved when the training set was balanced with resampling (except for MLP). The poor specificity caused the bad G-mean result of KNN and SVM in approach 1. Figure 4 presented that the KNN classifier obtained the optimal result with the AUC (0.58, SD = 0.01) and G-mean (55.70, SD = 3.04) after resampling. Moreover, the classification performance of KNN classifier got significant promotion after resampling in approach 2 compared with the case in approach 1. The reason can be explained by the introduction of synthesized data, which would be useful to build the feature space in the view of Euclidean distance.

**Table 2:** The parameter setting, sensitivity, and specificity of five classifiers in approach 1 and approach 2. Abbreviations: Sensitivity and specificity in approach 2 are expressed as mean ± standard deviation.

Classifier	Approach 1			Approach 2		
	Parameters	Sensitivity (%)	Specificity (%)	Parameters	Sensitivity (%) (mean ± SD)	Specificity (%) (mean ± SD)
KNN	K = 28	100	0	K = 2; 1; 2	76.09 ± 9.96	41.67 ± 9.55
RF	N = 215	91.30	18.75	N = 295; 155; 265	81.88 ± 3.32	12.50 ± 6.25
SVM	C = 1	100	0	C = 18; 29; 24	60.87 ± 40.73	47.92 ± 29.54
	gamma = 0.01			gamma = 0.3; 0.3; 0.3		
MLP	M = 5	60.87	37.5	M = 17; 66; 70	60.87 ± 2.17	33.33 ± 3.61
PLS-DA	n = 2	91.30	12.5	n = 6; 8; 5	63.05 ± 3.76	43.75 ± 6.25

### The classification performance of lung cancer subtypes with dimensionality reduction

**Figure 5:**
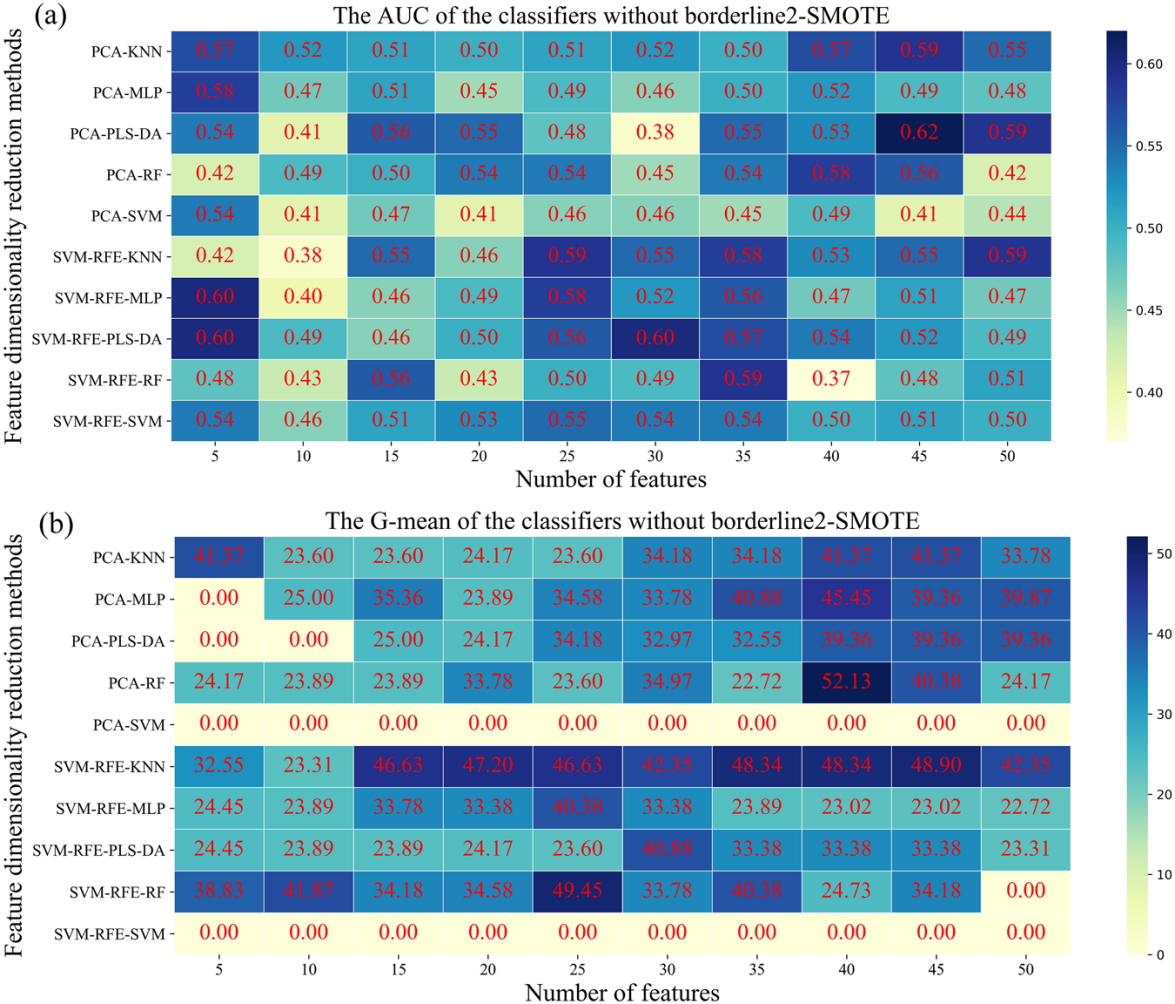
Heat map presents the predictive performance in approach 3. Five classifiers across two feature dimensionality reduction methods (in rows) and selected ranges (in columns) in adenocarcinoma and squamous cell carcinoma patients are presented. (**a**,**b**) Are the AUC and G-mean values of five classifiers without borderline2-SMOTE, respectively.

Given the redundant information that might make the classification results of SCC and AC unsatisfied in breathomics data, the feature dimension reduction methods, namely PCA and SVM-RFE, were adopted to retain relevant information and deduct irrelevant information. For each feature reduction method, we incrementally selected 5–50 features with five steps according to the importance of ranking as input data to classifiers. Figure 5 showed the classification performance of five classifiers in lung cancer subtypes on the testing set. The heat map in Fig. 5(a) showed that PCA-PLS-DA had the best performance (AUC = 0.62) when the number of breathomics features selected was 45. SVM-RFE-PLS-DA (AUC = 0.60), SVM-RFE-MLP (AUC = 0.60), got the same results, which indicated the importance of features in the phase of model training. Figure 5(b) showed that PCA-RF achieved the best G-mean (52.13) among 100 classification models, while its AUC value (0.58) was not the highest. SVM classifier obtained the worst classification performance both in AUC and G-mean. We guessed that the classification hyperplane interface had more AC samples resulting in the lousy specificity in the SVM classifier. Although PCA-PLS-DA had a better AUC value, G-mean (39.36) was not an ideal result for the classification of AC and SCC patients. Thus, the G-mean needed to be improved for better prediction performance in the classification model.

### The classification performance of lung cancer subtypes combined with resampling and dimensionality reduction

**Figure 6:**
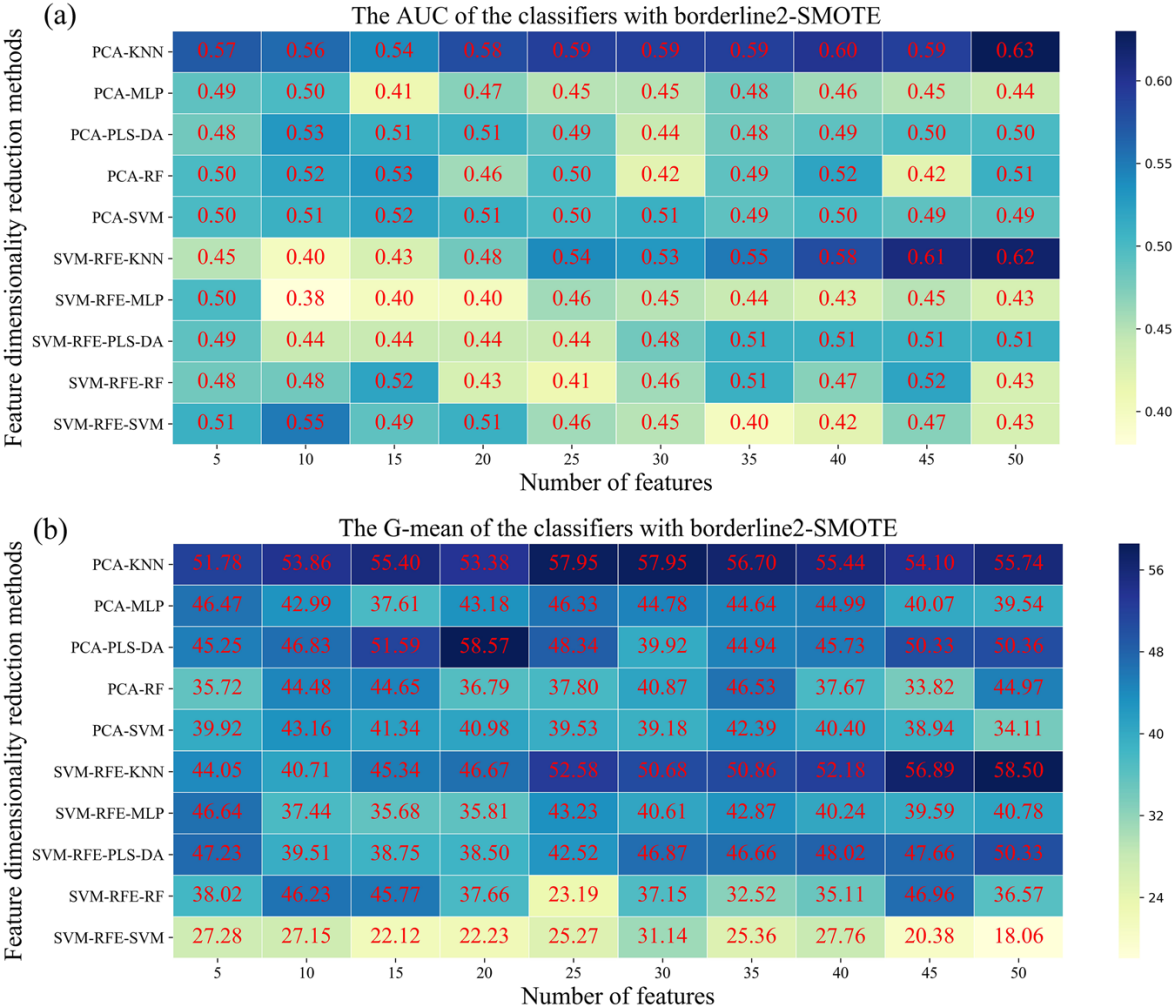
Heat map presents the predictive performance in approach 4. Five classifiers across two feature dimensionality reduction methods (in rows) and selected ranges (in columns) in adenocarcinoma and squamous cell carcinoma patients are presented. (**a**,**b**) Are the AUC and G-mean values of five classifiers with borderline2-SMOTE, respectively.

We further evaluated the performance of five classifiers in the classification of AC and SCC patients with borderline 2-SMOTE and feature reduction methods in approach 4. The average classification performance of three times in the 100 classification models based on the selected features was shown in Fig. 6. The result showed that PCA-KNN (AUC = 0.63, SD = 0.03) and SVM-RFE-KNN (G-mean = 58.50, SD = 1.79) had the highest predictive performance with 50 breathomics features and the KNN classifier was the least sensitive due to the tiny standard deviation in AUC and G-mean value. The PLS-DA based on the SVM-RFE method got the lowest average AUC (0.50) with 5 selected features. SVM, RF, and MLP got the similar result, and their AUC were increased slightly (average: 0.55; 0.53; 0.53) when compared to approach 1. Nevertheless, the specificity for all classifiers was improved from the original 0~35% to the range of 8~85% (Fig. S1), resulting in the increased G-mean value indirectly. The classification model paid more attention to the minority class (SCC) samples after resampling; thus its positive rate, namely sensitivity (Fig. S2) in the model, was decreased to some extent.

## Discussion

In this breathomics-based classification task, we explored different supervised classifiers combined with borderline2-SMOTE and dimensionality reduction methods on the classification of AC and SCC with an unbalanced class of exhaled breath samples. The results of five supervised classifiers based on four data processing approaches were discussed. AUC and G-mean values were selected as critical indicators to evaluate the performance of classification models. The results in this study showed that the KNN classifier combining with borderline2-SMOTE and PCA and SVM-RFE feature reduction method obtained the best performance in distinguishing AC from SCC patients. Although the other four classifiers achieved the best AUC with feature reduction methods, they were worse than the KNN classifier. We found that combining feature reduction with borderline2-SMOTE improved the G-mean compared with the other three data processing approaches.

The significant difference between approach 1, 2, 3, and 4 was whether the borederline2-SMOTE and the features reduction were performed during the data processing phase. Given the linear relationship between the newly synthesized minority samples and the original minority samples, the borderline2-SMOTE only was applied in the training set, not the whole dataset to secure that the similar feature of test data would not show in the training phase before fed into the model. The result in Table 2 and Fig. S1 showed that the specificity in classification models was increased in approach 2 and approach 4, which meant the more SCC samples were correctly classified and this result was consistent with the literature^37^. SVM-RFE and PCA would select different feature information as an input to classifiers resulting in different classification results, which proved that no single feature reduction method fits all classifiers. Figures 5 and 6 showed that classifiers rely on the key breathomics features who are essential to construct a model in the training phase. Moreover, the key breathomics features in our work could be identified. In 2012, Santonico *et al*. combined more performant breath sampling technologies, such as EBS sampling method, with electronic nose analysis achieving 75% accuracy for prediction^16^. At the same time, they employed the GC-MS and EBS sampling method for classification analysis and proved the good discrimination between the AC and SCC patients using PLS-DA model. Nevertheless, in the electronic nose analysis, the VOCs composing these breath fingerprints were beyond the scope of their study because of the theory of electronic nose technique. In the GC-MS analysis, they did not give specific classification results. Besides, the complexity of sampling method limited its development in the wider population.

In order to select key respiratory omics features, we used svm-rfe for recursive selection of feature subsets, instead of PCA, which needed to determine the number of components to obtain more data information. We found that classifiers based on the SVM-RFE method needed a few features to obtain the optimal AUC value in unbalanced data compared to the PCA-based method in balanced data (except for KNN). However, the unbalanced data in approach 3 obtained the worst G-mean value when compared to the balanced data in approach 4. For those phenomena, we suspected that the increased minority class samples changed the data distribution in the classification hyperplane of different classifiers. Thus, our training model based on balanced data could improve the prediction ability (sensitivity) of the minority class in the testing set.

In our work, the combined KNN classifier obtained the optimal classification result, and its classification performance was significantly changed after resampling SCC samples and reducing the irrelevant information. Due to the principle of KNN classifier, the classification result mainly depends on the number of neighbors selected, which is similar to borderline 2-SMOTE, that the generation of new data relies on the determination of adjacent samples of dangerous samples. Thus, most of the nearest neighbors are newly synthetic samples, and the model would be more robust than before for KNN classier. As for other classifiers, PLS-DA classifier performed better than the other three classifiers without considering the feature numbers because new features in PLS-DA were transformed linearly from original breathomics features. That is the reason why it is a popular way for omics analysis at present. Besides, PLS-DA needs to conduct model validation to avoid the over-fitting problem^19^. In contrast, KNN sets fewer parameters and can obtain better results quickly, which proves it much more suitable for classification tasks. The theory of SVM is to find a hyperplane that can separate different class samples depending on the support vector samples. When the data is not balanced, SVM has the following shortcomings. On the one hand, SVM is based on the soft interval maximization method, which makes the classification hyperplane in the boundary region towards to minority class. On the other hand, the unbalanced ratio of support vectors would also result in more majority support vectors around the testing set. The classification performance of SVM has been improved after undergoing the borderline2-SMOTE by comparing Figs. 4 and 6. RF is a relatively robust model, which reduces the overall variance of the model by gathering the results of multiple decision trees. With the increase of samples, more accurate classification results can be obtained by increasing the number of estimators of RF classifier. When the data is unbalanced, the MLP performance of single-layer network structure is better than other classifiers. However, once the data in the classification model is increased, the single-layer network structure cannot meet the classification requirements, and the multi-layer network MLP is the key to improve the classification performance.

Although our study on the exhaled breath of lung cancer subtypes patients obtained the best AUC result (AUC = 0.63, SD = 0.03) and G-mean (G-mean = 58.50, SD = 1.79) based on the combined KNN classifier, the diagnose ability was insufficient for clinical decisions. The reasons for the unsatisfactory classification result can be explained by one or by a combination of the following possible reasons: (1) Significant different compounds released by AC and SCC cells can be detected at the cellular level, but the exhaled breath concentration may be diluted through the human body. Thus, the final concentration of the human exhaled breath may be lower than the detection limit of GC-MS due to the limitations of exhaled breath collection and analysis method^8^. (2) Clinical demographic factors (Table 1), and other omics features would be important factors for the enhancement of the classification model. The previous study showed that body mass index (BMI) was negatively correlated with SCC patients, but for AC patients, the association was positive^52^. In future studies, another advanced exhaled breath collection and enrichment method should be considered to expand the range of detection. In addition, the performance of classifiers can be enhanced if we incorporate genomics features, proteomics features, and clinical features like tumor grade, location, smoking history, and BMI.

## Conclusions

In this paper, the application of breathomics analysis in the classification of AC and SCC patients was discussed through different combinations of feature dimensionality reduction methods, borederline2-SMOTE, and supervised classifiers. The results showed that the proposed KNN algorithms combining PCA with borderline2-SMOTE showed improved performance for the classification of lung cancer subtypes. These important breathomics features in exhaled breath were unearthed through feature reduction analysis and can be further recognized and used for metabolic pathway analysis. In addition, it is worth mentioning that in our work, we systematically presented the diagnose performance, sensitivity and specificity of each classification model after implementing resampling and/or dimensionality reduction. In summary, The results of this study should be considered as promising and able to provide direction for the design of future trials. In our future research, we will integrate multiple omics data for better diagnose ability.

## Supplementary information

Supplemental materials.

## Data Availability

The data used in this study are available from the corresponding author on reasonable request.
