# Supplementary material for: Exploratory study on classification of lung cancer subtypes through a combined K-nearest neighbor classifier in breathomics
Source: Sci Rep. 2020 Apr 3;10:5880. doi: 10.1038/s41598-020-62803-4 (PMC7125212; doi:10.1038/s41598-020-62803-4)
Supplement: Supplementary file 1 — Supplemental materials. [file 41598_2020_62803_MOESM1_ESM.pdf]

Exploratory study on classification of lung cancer subtypes through a combined K-nearest neighbor classifier in breathomics

Authors:

Chunyan Wang;

Yijing Long;

Wenwen Li;

Wei Dai;

Shaohua Xie;

Yuanling Liu;

Yinchenxi Zhang;

Mingxin Liu;

Yonghui Tian*;

Qiang Li*;

Yixiang Duan*.

Supplemental materials:

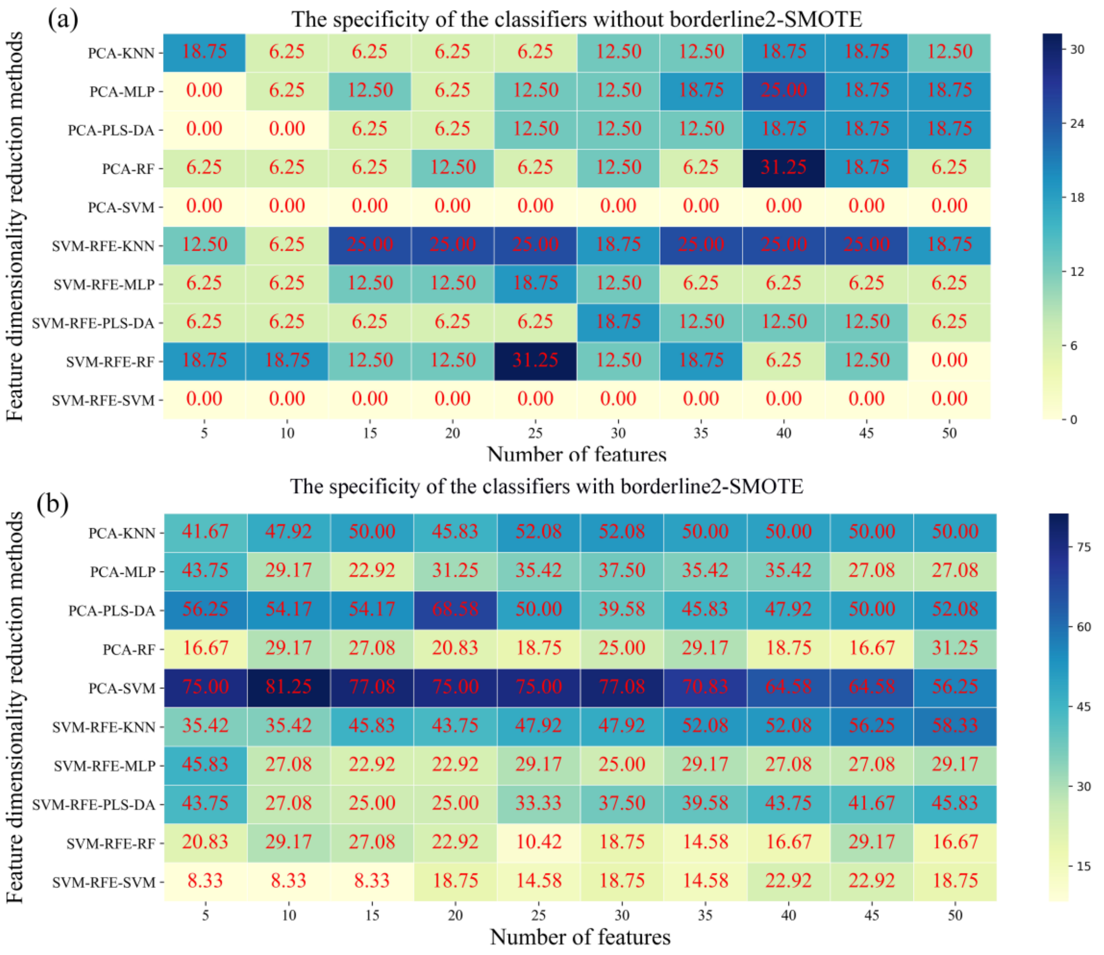

Figure S1. Heat map presents the specificity value of five classifiers before (a) and after (b) using borderline2-SMOTE. Five classifiers across two feature dimensionality reduction methods (in rows) and selected ranges (in columns) in lung cancer histological subtypes are presented.

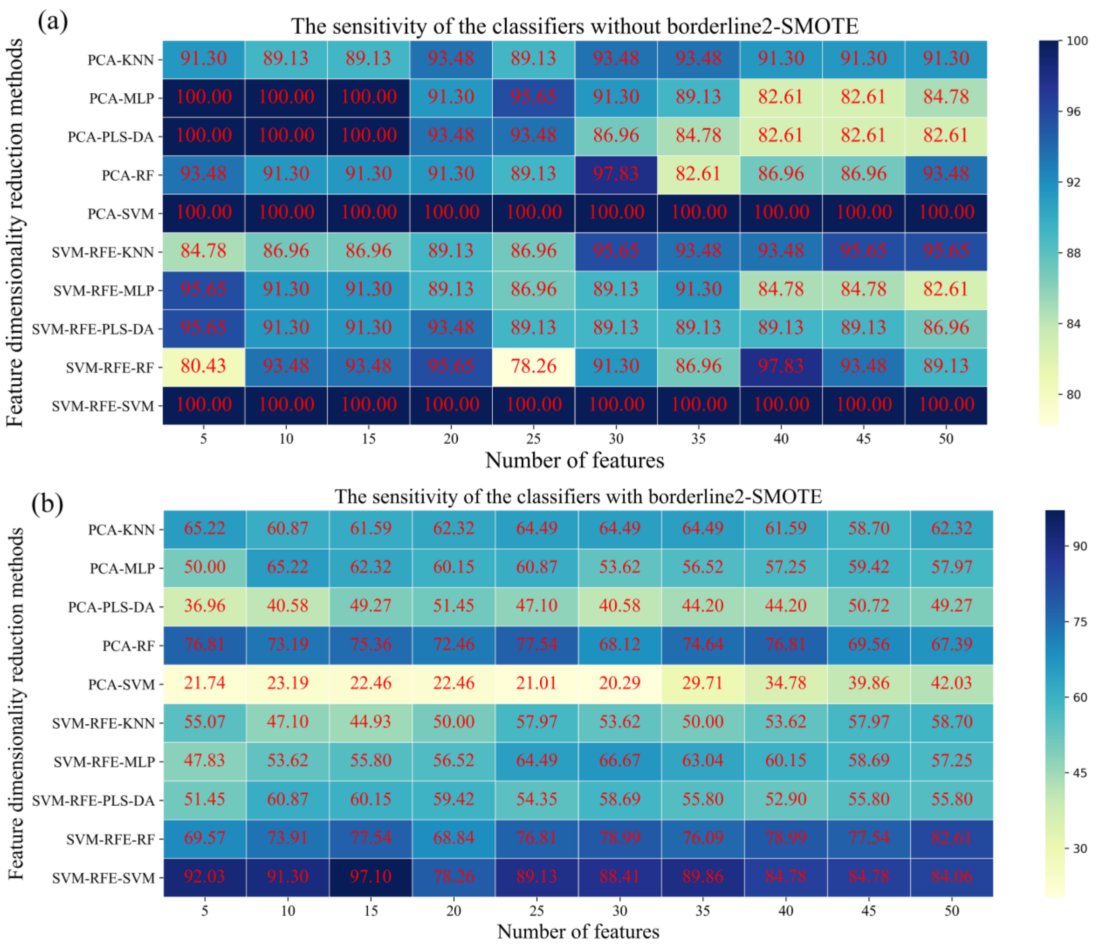

Figure S2. Heat map presents the sensitivity value of five classifiers before (a) and after (b) using borderline2-SMOTE. Five classifiers across two feature dimensionality reduction methods (in rows) and selected ranges (in columns) in lung cancer histological subtypes are presented.
